# Modifying Water–Frost Resistance and Mechanical Properties of Lime Mortar Using Siliceous and Fluidised Bed Fly Combusted Ashes Activated with Cement

**DOI:** 10.3390/ma16083013

**Published:** 2023-04-11

**Authors:** Dominik Logoń, Janusz Kobaka, Jacek Domski

**Affiliations:** 1 Faculty of Civil Engineering, Wroclaw University of Science and Technology, Wybrzeże Wyspianskiego 27, 50-370 Wroclaw, Poland; 2 Faculty of Geoengineering, University of Warmia and Mazury in Olsztyn, Prawochenskiego Street 15, 10-720 Olsztyn, Poland; 3 Faculty of Civil Engineering, Environmental and Geodetic Sciences, Koszalin University of Technology, Śniadeckich Street 2, 75-453 Koszalin, Poland

**Keywords:** lime mortar, siliceous and fluidised bed fly ashes, cement, frost resistance, mechanical properties

## Abstract

The research focuses on pozzolanic additives, which are compatible with traditional lime mortars, and enable the modification of the rheological, physical and mechanical properties of tested composites. It was noted that lime mortars with fluidised bed fly ash require sand without impurities to avoid possible ettringite crystallisation. The work presents siliceous fly ash and fluidised bed combustion fly ash to modify the frost resistance and mechanical properties of traditional lime mortars with and without the addition of cement. The results show better effects using fluidised bed ash. Traditional Portland cement CEM I 42.5R was used to activate ash and increase the results. The possibility of a significant improvement of properties is indicated with a hybrid addition to the lime binder of 15–30% ash (siliceous or fluidised bed ash) and 15–30% cement. Changing the class and type of cement provides an additional opportunity to alter the properties of the composites. For architectural reasons relating to colour, the suitability of lighter fluidised bed ash instead of darker siliceous ash and of white Portland cement instead of the traditional grey cement can be used. The proposed mortars can be the basis for future modifications with admixtures and additives, e.g., metakaolin, polymers, fibres, slag, glass powder and impregnating agents.

## 1. Introduction

The oldest finds indicating the use of lime in various structures around the world date back to around the 4–10th century BC (e.g., lime plasters in Ain Ghazal in Jordan and Çatalhöyük, Turkey 7500 BC; lime mortars in the Egyptian pyramids 4000 BC). For thousands of years, humans have been modifying mortars with various admixtures and additives to improve their physical and mechanical properties [1,2,3,4]. Some of the oldest additives include, for instance, diatomaceous earth, clay, bristles, animal blood, horsehair, slag and ashes including volcanic tuffs. Studies of historic lime mortars also show their modification with the addition of wood-burning ash [5] also modern publications take into account the influence of organic ash [6,7].

Much of the current research focuses on the addition of cement and different kinds of ash of varying compositions [8,9,10,11,12,13,14]. In addition, other additives are commonly used, such as, e.g., metakaolin [15], glass and brick powder [16], slags and impregnating agents including those based on silanes [17,18,19,20,21].

The production of cement and lime is characterised by high CO_2_ emissions and high- energy consumption [22,23]. Modern environmental conditions necessitate the reduction of greenhouse gas emissions and the use of additives helping to reduce emissions of these gases. Granulated blast furnace slag and siliceous fly ash are the best known and widely used Portland clinker supplements [24,25,26,27,28,29,30].

The use of fly ash made from organic materials (mainly biomass combustion) in the production of unreinforced concrete elements can be also taken into account. The main problem of using such ashes is to ensure a stable chemical composition. Further research is required to determine the application range. It seems that such ashes are not commonly used and are often based on regional technical specifications [31].

Siliceous fly ashes are the by-products obtained from the combustion of hard coal. Low-calcium fly ash obtained is a commonly used ash. The typical amount of siliceous fly ash in cement composites does not exceed 35% of the cement weight [29]. It is possible to use as binders high-volume fly ash 40–60% and 100% alkali-activated fly ash as well [30,31,32,33,34,35,36,37,38,39].

There are some requirements determining the quality of fly ash as a mineral addition to concrete [9]. The fly ash pozzolanic activity index depends on the alkali content in cement. This index becomes higher with a higher alkali amount in cement. The rate of cement substitution by fly ash depends on its cementing efficiency. The fly ash pozzolanic index is determined after 28 and 90 days and requirements for fly ash such as fineness and chemical composition have to be considered [31,33,40].

Fluidised bed fly combusted ashes are obtained from the combustion of brown coal. Different varieties of such ashes, such as high-calcium fly ash and fluidised bed fly ash are used to modify the properties of cement composites [41,42,43,44,45,46,47]. The possibility of using fluidised bed ash in these composites is limited due to the possibility of ettringite formation causing structural destruction [40,48,49].

It is notable that there is relatively less research using fluidised bed ash to modify properties of traditional lime mortars compared to siliceous ash applications (references). The reason for this may be the lower availability of fluidised bed ash as well as the greater difficulty to maintain a constant mineralogical composition during the combustion of brown coal in fluidised bed furnaces [31]. Significant differences in the mineral composition of those ashes contribute to problems with consistency, especially with the high content of these additives in mortars. Another problem is the possibility of gypsum formation, the primary cause of which is the high content of free lime in the composition of those ashes. Free CaO binds to SO_2_ which is a residue from the combustion of coal and occurs at the desulphurisation process, allowing gypsum to form CaSO_4_ [50]:

CaCO_3_ → CaO + CO_2_ (1)

SO_2_ + CaO + ½ O_2_ → CaSO_4_ (2)

If the CaSO_4_ sets too quickly compared to CaCO_3_, it can cause damage to the composite structure. Whereas the ettringite corrosion results in an increase in volume in cement composites [40,49] in the case of gypsum the destruction is caused by a faster setting process in lime mortars compared to other components and causes damages in the composite structure [50].

Sulphate corrosion occurs in solutions of sulphate salts, sulphuric acid and humid air containing SO_2_ as a result of the precipitation reaction of gypsum (calcium sulphate), as well as in direct contact between concrete and gypsum. Ettringite (Candlot’s salt) as a result of cement reaction (sulphate salts and tricalcium aluminate) tricalcium aluminate 3CaO·Al_2_O_3_·3CaSO_4_·32H_2_O (C_3_A·3CS·32H) is formed [40,49,51]. Ettringite increases its volume (approx. 168%) causing cracks and the destruction of cement composites.

In this paper, it was noticed that sand without impurities should be used to avoid possible ettringite crystallisation in lime mortars with the addition of fluidised bed combustion fly ash. The optimisation of fluidised and siliceous ash to improve the mechanical properties and water–frost resistance (which determines the durability of lime mortars) was performed. In addition, fluidised bed ash (more effective than siliceous ash) activated with cement was used, achieving a multiplication of the desired effects. It was noted that the extent of the modification of traditional lime mortars should be limited, and attention should be focused on increasing durability while maintaining the original characteristics of those composites’ properties including architectural qualities relating to colour. The research results presented in this paper offer the possibility of obtaining composites which can be further modified with hybrid admixtures and additives.

## 2. Materials and Methods

### 2.1. Materials

The following components were used for lime mortars:

L—hydrated lime consistent with EN 459-1: CL 90-S;
K—siliceous fly ash from hard coal combustion consistent with EN 450-1: (loss on ignition ≤ 5%);
F—fluidised bed ash from brown coal combustion; “Flubet”, Turów Power Station;
C—Portland cement CEM I 42.5R; Górażdże Cement plant;
P—quarz sand 0–2 mm; aggregate mine in Mietków;
W—tap water.

Table 1 shows the ranges of chemical compositions of siliceous and fluidised bed ash produced in Polish combined heat and power plants.

The specimens were moulded into 40 mm × 40 mm × 160 mm beams and kept in a humidity chamber for 28 days in relative humidity of 95% RH (nine specimens of each series). The specimens were left under laboratory conditions at 20 °C (next left in laboratory room). The samples were tested 90 days after moulding.

Table 2 presents the designations and compositions of the mortars used in the experiment. The referenced mortar M0 corresponds to a traditional lime mortar based on hydrated lime CL 90-S characterised by compressive strength of approximately 1 MPa. The other mortars were prepared with the addition of low-calcium siliceous ash (from the combustion of hard coal 30% of the lime weight—K30), fluidised ash (from the combustion of brown coal 30% of the lime weight—F30), a hybrid of 15% of cement and 15% of fluidised ash C15F15 and a hybrid of 30% of cement and 30% of fluidised ash C30F30.

Tap water was added in the amount that made it possible to obtain a constant mortar consistency (PN-B-04500 [52]; Novikov’s cone—consistency 6 cm).

### 2.2. Methods

#### 2.2.1. Microstructure: The Semi-Quantitative Analysis SEM-EDS and the Phase Analysis XRD

SEM (scanning electron microscope) measurements were made using Hitachi S-3400N scanning electron microscope equipped with a tungsten cathode. Images were recorded using electrons backwards scattered method. EDS (energy dispersive X-ray spectroscopy) measurements were made with a Noran System 7 analyser with a Thermo Scientific Ultra Dry detector with a resolution of 129 eV. SEM-EDS is a semi-quantitative analysis. The tests were conducted in a low vacuum at pressure of 50 Pa in a measuring chamber. All measurements were performed under tension of 30 kV. XRD (X-ray diffraction) was carried out on Aeris Panalytical 5-110, 2-theta. Phase analyses by XRD were used to determine the crystallographic structure of a material. It works by irradiating a material with incident X-rays and then measuring the intensities and scattering angles of the X-rays that leave the material.

#### 2.2.2. Water Absorption Test

The water absorption test was carried out by placing lime mortar beams (40 mm × 40 mm × 160 mm) in a vertical position in a cuvette filled with water to a height of 10 mm. The test was completed for all samples when the reference mortar M0 (showing the highest absorption) achieved capillary action to 2/3 of its height. It took an hour, and all samples were taken out of the water at the same time. The test results present water absorbed by the samples as a percent by volume.

#### 2.2.3. Water–Frost Resistance Test

The water–frost resistance test was conducted by the freezing and thawing cycles of the lime mortar beams’ halves. One cycle consisted in flooding the specimens with water and storing them at 20 °C for 4 h. Then the samples were extracted from water and frozen in a wet state at −20 °C for 4 h. One hour after the start of the next cycle (flooding with water), the specimens were extracted from water, visually assessed, weighed and documented photographically.

The specimens that were damaged, showed cracks or had a weight loss of more than 5% were considered to present a negative water–frost resistance test result.

This test was dedicated to frost resistance concrete, which is a hydraulic binder composite, PN-B-06265 [53]. In the case of air binder mortars, it is not used and therefore it was considered a test of water–frost resistance.

#### 2.2.4. Compressive Strength Test

The compressive strength test f_c_ was performed on the beam halves obtained from the three-point bending test. The compressive test was performed according to the guidelines for standard cement mortar [54].

#### 2.2.5. Three-Point Bending Test

The three-point bending test f_tb_ was performed according to the guidelines for standard cement mortar [54]. Bending tests were carried out in the testing machine with controlled displacement. (Beams 40 mm × 40 mm × 160 mm—spacing of supports 150 mm). The load–displacement curves were obtained based on the measurement of the load and constant displacement of crosshead.

## 3. Results

### 3.1. Microstructure: The Semi-Quantitative Analysis SEM-EDS and the Phase Analysis XRD

**Figure 1:**
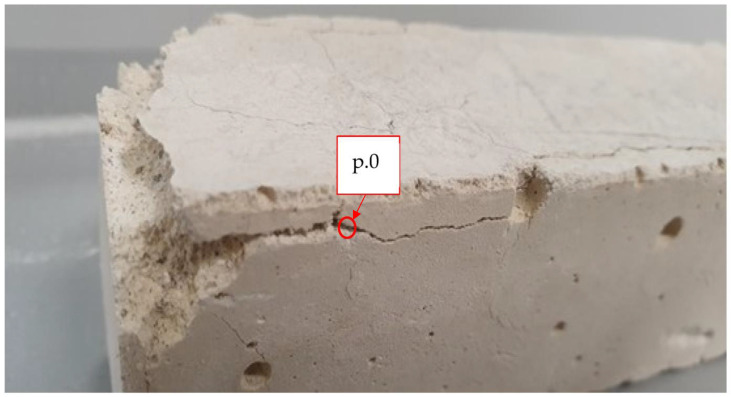
Self-destruction process of lime mortar with 30% fluidised bed ash, F30.

Figure 1 shows the self-destruction effect of lime mortar with a 30% addition of fluidised bed ash (specimen F30). The visible destruction process took place in one of the nine prepared specimens of one series after 28 days of storage in the humidity chamber. The other specimens showed no destruction effect. No additional damage (in the samples) was observed after 90 days before the physical and mechanical tests.

**Figure 2:**
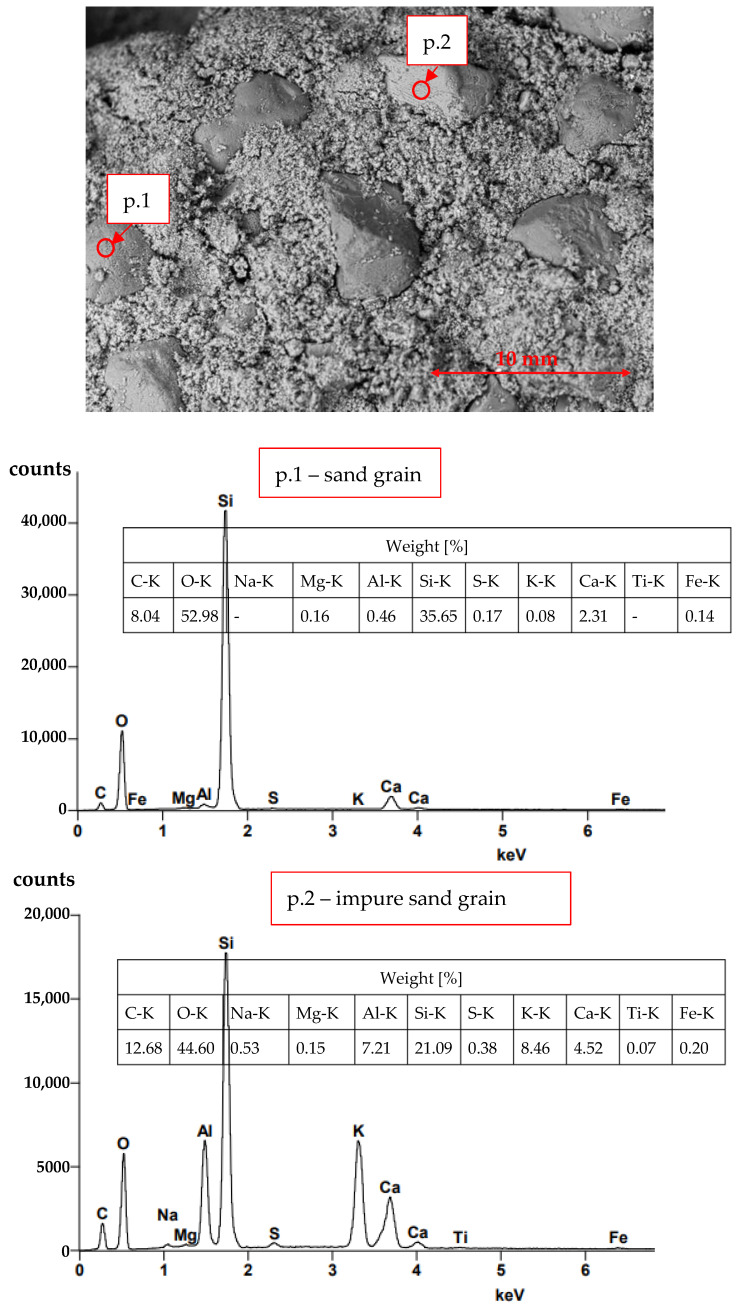
The semi-quantitative SEM-EDS analysis of self-destructed lime mortar with 30% fluidised fly bed ash, F30.

**Figure 3:**
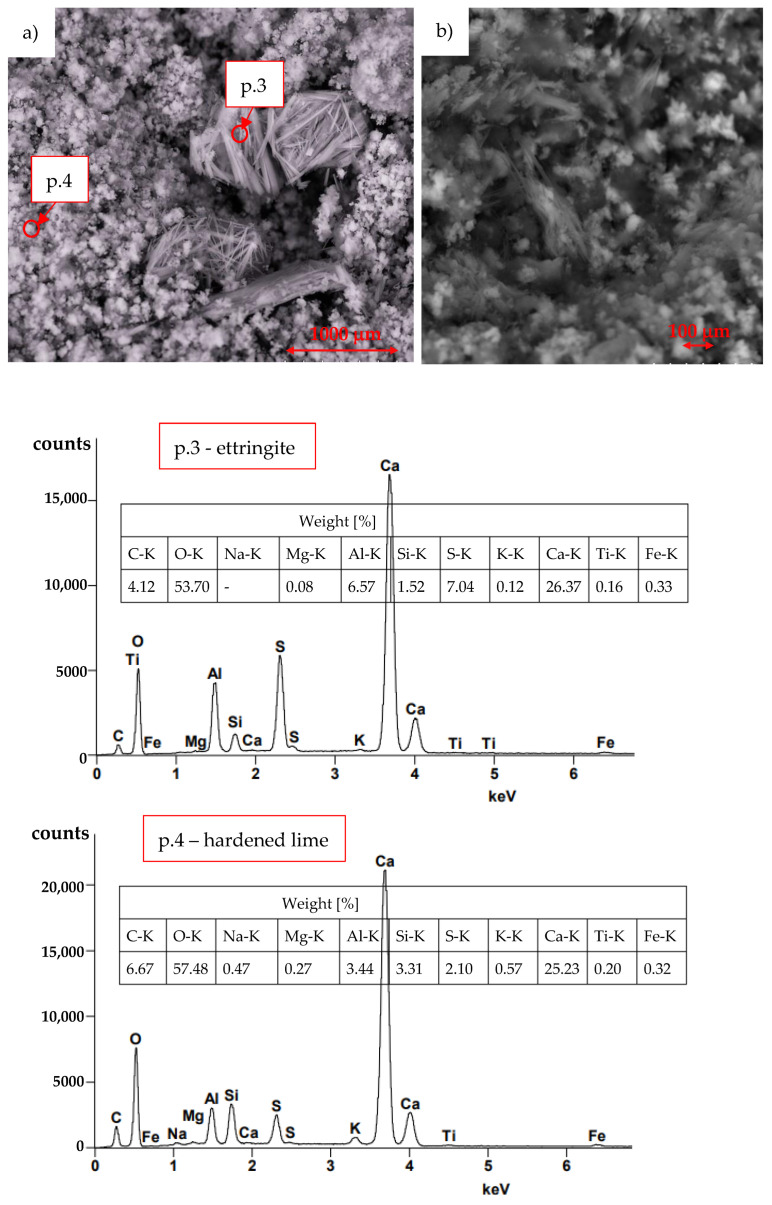
The semi-quantitative SEM-EDS analysis of self-destructed lime mortar with 30% fluidised bed ash, F30: (**a**) p.3 identification of ettringite concentration, p.4 structure of the hardened lime, (**b**) crystallisation of ettringite needles.

Figure 2 and Figure 3 present the semi-quantitative SEM-EDS results for the F30 mortar at the areas of the cracks. Figure 2 shows the image of lime mortar with sand grains. Point p.1 identifies a slightly impure grain of sand. Point p.2 identifies a significantly impure grain of sand. The content of identified elements is by weight (%).

Figure 3 shows areas where the self-destruction of the lime mortar structure occurred. Point p.3 in Figure 3a presents the concentration of ettringite crystallisation, and Figure 3b shows needles of ettringite crystallisation. Point p.4 presents a fragment of lime structure. For individual points, the identified elements were listed by weight (%).

**Figure 4:**
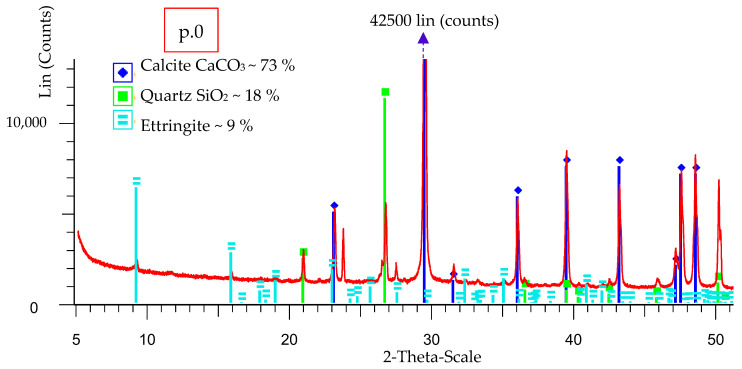
The X-ray phase analysis diffraction test of self-destructed lime mortar with 30% fluidised bed ash, F30.

Point p.0 (Figure 1) shows the place of the X-ray phase analysis diffraction test. The results of the XRD analysis are presented in Figure 4.

The semi-quantitative analysis SEM-EDS and the phase analysis XRD were tested in many places presenting only the most significant ones.

Figure 4 shows the X-ray phase analysis diffraction test of mortar F30 in the crack area at point p.0 (Figure 1). Based on the analysis the presence of calcite CaCO_3_ quartz SiO_2_ and ettringite was found.

The obtained results of SEM-EDS and XRD confirm the presence of ettringite in places of cracks in the lime mortar F30.

### 3.2. Water Absorption Test and Water–Frost Resistance Test

The test results of water absorption and the number of water–frost resistance cycles of the lime mortars are summarised in Table 3.

**Figure 5:**
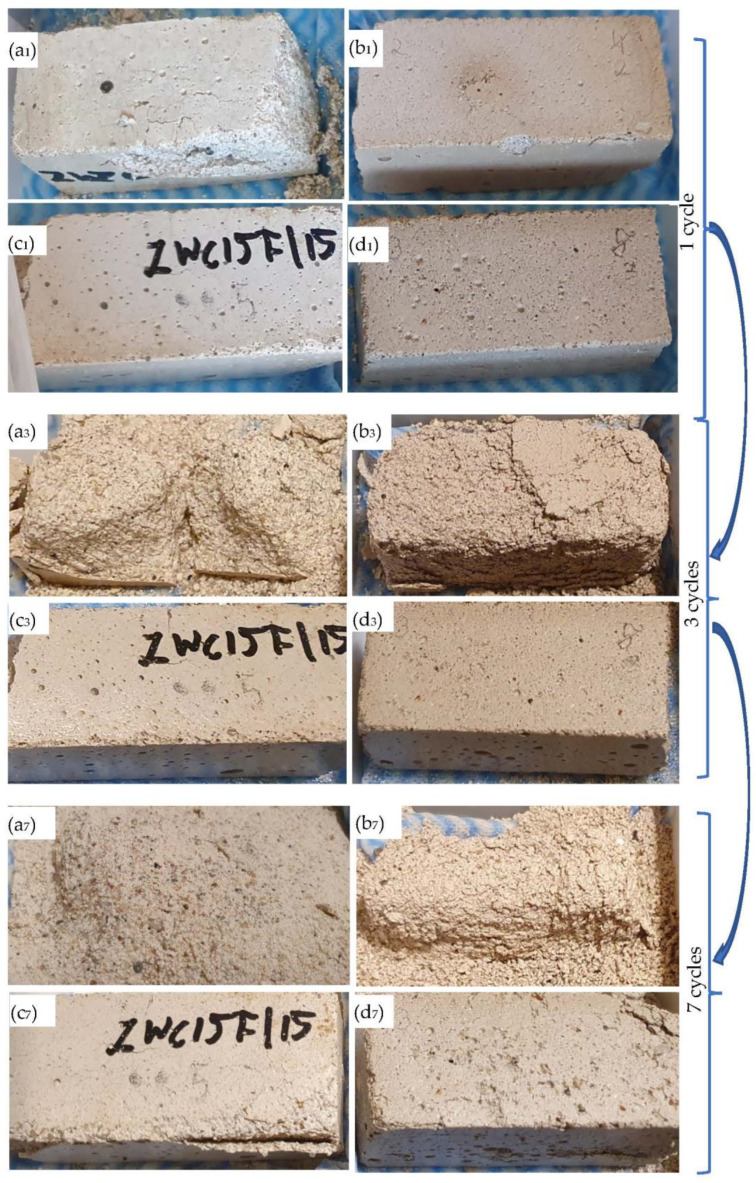
General view of lime mortar specimens after 1, 3 and 7 cycles of water–frost resistance test: (**a_1,3,7_**) M0; (**b_1,3,7_**) F30; (**c_1,3,7_**) C15F15; (**d_1,3,7_**) C30F30.

Figure 5 presents the examples of the tested beam halves lime mortars composites after 1, 3 and 7 cycles of the water–frost resistance tests. The photos show samples after the start of another cycle—1 h after flooding the specimens with water. Subsequently, they were placed into the water to continue with the next cycle. The presented photographs of mortars were taken during the day under natural light (after cycle 1) and in the evening under artificial light (after cycles 3 and 7), which has a slight effect on the colour of the surface of the specimens.

The water–frost resistance test should be considered a test of the hybrid effect of water resistance and frost resistance (water–frost resistance test). The procedure was not established for lime mortars which are considered to be air binder mortars, but it shows well the effectiveness of modifying the resistance of lime composites to water and frost, see Figure 5.

The specimen made of reference lime mortar M0 degraded after one cycle of water–frost resistance test (see Figure 5a_1,3,7_). The specimens K30 (not presented in Figure 5) and F30 (see Figure 5b_1,3,7_) were damaged after one and two cycles, respectively. The best water–frost resistance was achieved in lime mortars C15F15 and C30F30, respectively, after five and six cycles (see Figure 5c_1,3,7_,d_1,3,7_).

### 3.3. Mechanical Tests

Table 4 summarises the strength parameters of the tested mortars; compressive strength f_c_, three-point bending tensile strength f_tb_, brittleness k = f_c_/f_tb_, tgα_c_ and tgα_tb_ (slope angle of the load–crosshead displacement curve in f_c_ and f_tb_ tests). In addition, the amount of absorbed energy understood as the area under the load–displacement of crosshead curve W_c_ and W_tb_, respectively, was determined. The results of physical and mechanical properties were presented as an arithmetic mean of three specimens (relative error does not exceed 13%).

**Figure 6:**
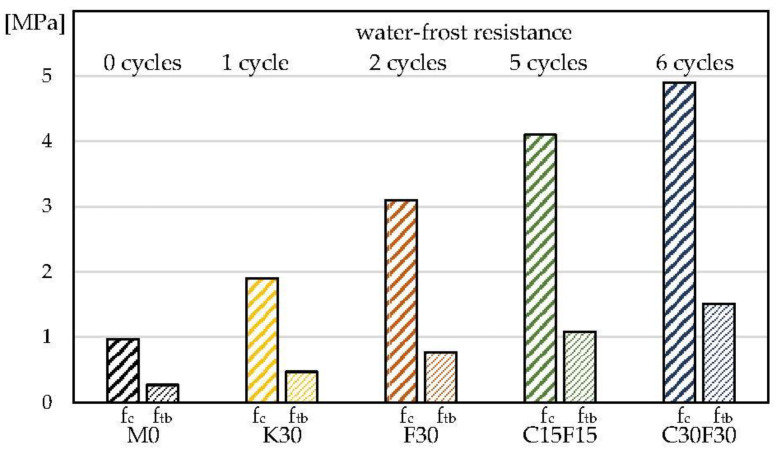
The compressive strength f_c_, three-point bending tensile strength f_tb_ and water–frost resistance of the tested lime mortars.

Figure 6 summarises the compressive strength f_c_, three-point bending strength f_tb_ and water–frost resistance of the tested composites.

**Figure 7:**
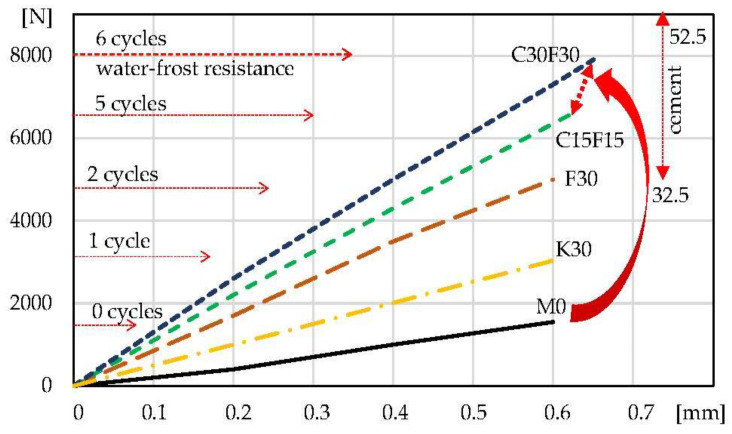
Load–displacement of crosshead curves: lime mortars during compression f_c_ with water–frost resistance results.

**Figure 8:**
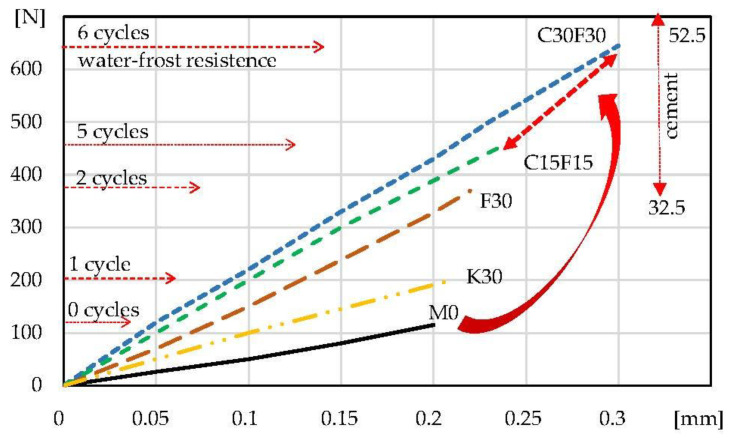
Load–displacement of crosshead curves: lime mortars during tensile bending f_tb_ with water–frost resistance results.

Figure 7 and Figure 8 show the composites under load during the f_c_ and f_tb_ tests. The load–displacement of crosshead correlation enables the determination of the tgα, which indicates the effect of the additives used on the change in the deformation ability of the tested composites. In addition, information on the assumed effect of changing the class of cement on the properties of composites with C15F15 and C30F30 hybrid additives is in the upper right corner. The information about the corresponding water resistance test is on the left side.

Table 4 and Figure 5, Figure 6, Figure 7 and Figure 8 present that the 30% addition of siliceous ash improved the physical and mechanical properties of K30 mortar approximately twice compared to M0. Specimens F30 with 30% fluidised bed ash addition showed an improvement (approx. two times) in properties compared to mortar K30 with siliceous ash addition. Further tests continued with a hybrid addition of fluidised bed ash and cement. Modified C15F15 and C30F30 lime mortars presented the best results in improving the tested properties. These composites are also characterised by the largest amount of absorbed energy in the destruction process (W_c_, W_tb_—Table 4).

## 4. Discussion

The conducted tests aimed at increasing the durability of traditional lime mortars. They were proposed to modify the composition of mortars with the addition of low-calcium siliceous ash, fluidised bed ash and Portland cement CEM I 42.5R. The discrepancies in the compositions of fluidised bed ash obtained from brown coal combustion [31] mean that the results obtained from testing this additive cannot be generalised and should be verified each time the ash producer is changed. Another reason for the limited applicability of fluidised bed combustion ash is the problem of ettringite formation in cement composites, which increases in volume [40,48]. Porous lime mortar with a small addition of cement limits the negative impact of this effect.

Free CaO binds to SO_2_, which is a residue from the combustion of coal and occurs at the desulphurisation process, allowing gypsum to form CaSO_4_ (reactions 1 and 2). That possible reaction (described in the literature [50]) proceeds faster than in the case of other mortar components, generating stresses in the composite structure. To reduce possible self-destruction effect (gypsum concentration) a cement additive is recommended to regulate the setting time.

The self-destruction process occurred in one of the nine MF30 specimens (see Figure 1) in a mortar with the addition of 30% of fluidised bed ash. The semi-quantitative SEM-EDS analysis shows the identification of elements on sand grains at points p.1 and p.2, Figure 2 (mortar MF30, Figure 1—in place of self-destruction). The sand grain (Si identification) at point p.1 shows a smaller number of impurities than the sand grain at point p.2. Particularly noteworthy is the increased content of Al on the sand grain in point p.2 which is needed for the crystallisation of ettringite 3CaO·Al_2_O_3_·3CaSO4·32H_2_O. The results summarised in Figure 3a show the concentration of ettringite crystallisation at point p.3 and the significant content of Ca, O and Al, S. Al and S are commonly found in soils. At point p.4 of the structure of the hardened lime (where there is no visible crystallisation), the occurrence of Al and S is lower. Figure 3b shows scattered needles of ettringite crystallisation. The X-ray phase analysis diffraction test identifies the presence of CaCO_3_, SiO_2_ and ettringite, Figure 4. The conducted SEM-EDS and XRD analysis confirmed the presence of ettringite as the cause of the self-destruction of the MF30 mortar structure presented in Figure 1. The obtained results indicate that the direct cause of the occurrence of ettringite was the concentration of impurities in the sand used for the study. The random inclusion of impurities in the sand explains why only one of the tested samples showed the process of self-destruction. It is recommended to use clean standard sand or washed sand free of impurities to avoid the uncontrollable formation of ettringite.

Table 3 shows that there is no significant increase in absorbed water (samples with the addition of ash and cement). Ash and cement were added to the composition rather than replacing part of the lime, which may have influenced the results. It can be assumed that replacing part of the lime, especially with fluidised bed ash and cement, will result in a slight reduction in absorbed water. The obtained results indicate the need for impregnation of the test specimens if such assumptions are required. Impregnation can take place at the composite forming stage (e.g., with polymers) or at the surface after application (e.g., with silane-based impregnating agents).

As can be seen on the basis of Figure 5a_1_, the reference lime mortar M0 was damaged after one cycle, and after seven cycles individual sand grains were separated, Figure 5a_7_. The 30% addition of siliceous ash (sample K30) increased the water–frost resistance slightly to one cycle (see Table 3). The 30% addition of fluidised bed ash (sample F30) increased the water–frost resistance to two cycles. Better results for water–frost resistance and mechanical properties were obtained with the addition of fluidised bed ash (compared to siliceous ash)—further activation of the properties with cement was carried out for F30 mortars. The analysis of the results indicates that the water–frost resistance of traditional lime mortar can be significantly improved from zero cycles to six cycles with the addition of 30% fluidised bed ash and 30% of cement, C30F30. The destruction process (due to water–frost exposure) in lime mortars without and with the addition of ash is characterised by significant surface destruction, while the higher-strength composites with the addition of cement (C15F15 and C30F30) undergo a similar process with additional observable cracks in the structure, see Figure 5.

Traditional M0 lime mortar characterised by a compressive strength of approximately 1 MPa does not show sufficient durability. Sample M0 is a very weak and brittle composite that deteriorated after one freeze–thaw cycle.

As can be seen from Figure 5, Figure 6, Figure 7 and Figure 8 and Table 4, the addition of 30% siliceous ash K30 and 30% fluidised bed ash F30 enables a significant improvement in the mechanical properties and water–frost resistance of lime mortars. The results show more than two times better effects with the use of fluidised bed ash compared to siliceous ash, which is why the focus of the subsequent testing was on a hybrid 15–30% fluidised bed ash addition and 15–30% cement addition (C15F15 and C30F30). The reasons for better results of lime mortars with the use of fluidised bed fly ash compared to siliceous fly ash can be correlated with a higher consolidated microstructure which is confirmed by the results of lower water absorption (compare K30 and F30, Table 3). The explanation of this phenomenon requires separate analyses and tests.

The best results (water–frost resistance, f_c_, f_tb_, tgα, amount of absorbed energy W) were obtained for lime mortar with a maximum of 30% addition of fluidised bed ash and 30% cement, C30F30. A comparison of the brittleness (k, Table 4) of the tested mortars shows that the ash and cement additives have no significant effect on their brittleness.

The presented tests (Figure 7 and Figure 8, the load–displacement of crosshead) show that the modification of traditional lime mortars does not reduce the deformation capacity and slightly increases when activated with cement. In order to test the deformability of composites a different type of test is provided. It is important for mortars to ensure the deformation of structural elements (e.g., in brick walls). Absorbed energy (Table 4) shows that the modifications of lime mortars significantly increase W_c_, and W_tb_ during the destruction process.

The properties of the mortars varied when modified with Portland cement. Changing the cement to class 32.5 or 52.5 enabled additional adjustment of mechanical properties, including water–frost resistance, depending on the assumptions made. The expected effect on the physical and mechanical properties was indicated in the upper right corner in Figure 6 and Figure 7.

Changing the class of cement will allow adjustment (increase/decrease in strength). In traditional low-strength mortars, too much strength can be considered a defect. Changing the type of cement (e.g., with a high content of slags) will increase water resistance.

The acceptable extent of the modification of lime mortars remains debatable. In this paper, additives that increased strength parameters were used (including water–frost resistance) by about 5–6 times. It seems that the proposed number of additives and the extent of mechanical properties modification and water–frost resistance allow the modified composites to be considered as traditional lime mortar with increased parameters.

The fly ash and cement additives result in a slight change in the colour of the composites which can be a significant disadvantage in the conservation of historic monuments. This effect is more significant when the specimens are soaked in water. The fluidised bed combustion ash is lighter in colour than siliceous ash. The use of the amount of Portland cement representing 30% of cement weight remains a problem as this quantity of binder results in a grey hue to the specimens. In addition, 15% of fluidised bed combustion ash and 15% of cement do not significantly change the colour which appears to be acceptable. If the white–grey shades are not acceptable, traditional Portland cement can be replaced with white Portland cement to adjust the colours. Historical lime mortars (especially those that have been subject to modification [4]) often deviate from the white colour of pure lime. In such cases, the use of grey siliceous ash with the addition of ordinary Portland cement may be more justified.

## 5. Conclusions

On the basis of the conducted research, the following conclusions can be drawn:

(1) It is recommended to control the mineralogical composition of fluidised bed combustion fly ashes before their application in lime mortars to eliminate gypsum concentration and to use clean sand without impurities to eliminate the possibility of ettringite crystallisation.
(2) The improvement in water–frost resistance of lime mortars with the addition of siliceous ash and fluidised bed ash was noticed. The obtained results indicate that the verified addition of fluidised bed ash improves water–frost resistance and strength parameters.
(3) The effective addition of ash to lime mortars to improve their physical and mechanical properties should be 15–30% of the lime weight, and the water–frost resistance and the mechanical parameters can be activated by 15–30% cement content (also by its type and class).
(4) Cement and ash additives representing 30% of lime weight affect the colour of traditional lime mortar, so (for architectural reasons) using white cement together with the addition of fluidised bed ash should be considered.
(5) The obtained results of lime composite tests may be the basis for further modifications with various admixtures and additives increasing their durability.

## Figures and Tables

**Table 1:** Chemical—of ashes from Polish thermal power plants [31].

Component	Siliceous Fly Ash K (%)	Fluidised Fly Ash F (%)
LOI	1.8–8.5	1.0–10.0
SiO_2_	47.7–51.0	33.0–50.0
Al_2_O_3_	20.0–28.6	13.0–32.0
Fe_2_O_3_	5.8–8.8	5.0–10.0
CaO	3.6–6.2	8.0–18.0
CaO free	-	2.8–4.7
MgO	2.2–4.8	1.0–2.5
SO_3_	0.2–0.7	3.0–12.0
Na_2_O	0.9–1.3	0.4–3.0
K_2_O	2.2–3.5	1.0–4.0
Cl^-^	0.01	n.a.

**Table 2:** Lime mortars used in the research.

Symbol	Proportion of Mortar Components	Weight Proportion
M0	lime:sand	1:4.5
K30	lime:sand:siliceous ash	1:4.5:0.3
F30	lime:sand:fluidised bed ash	1:4.5:0.3
C15F15	lime:sand:fluidised bed ash:cement	1:4.5:0.15:0.15
C30F30	lime:sand:fluidised bed ash:cement	1:4.5:0.3:0.3

**Table 3:** The amount of water absorbed and water–frost resistance of tested lime mortars.

Symbol	Water Absorption—1 h Volume (%)	Water–Frost Resistance
M0	16.00	0 cycles—surface destruction
K30	18.22	1 cycle—surface destruction
F30	17.64	2 cycles—surface destruction
C15F15	18.80	5 cycles—cracks/surface destruction
C30F30	19.98	6 cycles—cracks/surface destruction

**Table 4:** Strength parameters of tested lime mortars.

Symbol	f_c_ [MPa]	f_tb_ [MPa]	k = f_tb_/f_c_	tgα_c_	tgα_tb_	W_c_ [kJ]	W_tb_ [kJ]
M0	0.97	0.27	0.28	2583	575	465	11.50
K30	1.9	0.47	0.25	5067	952	912	21.00
F30	3.1	0.77	0.25	8333	1682	1500	40.70
C15F15	4.1	1.08	0.26	10,581	1917	2034	55.20
C30F30	4.9	1.51	0.31	12,154	2150	2567	96.75

## Data Availability

Not applicable.
